# Supplementary material for: Management and outcome of post-myocardial infarction ventricular septal rupture—A Low-Middle-Income Country Experience
Source: PLoS One. 2022 Oct 27;17(10):e0276615. doi: 10.1371/journal.pone.0276615 (PMC9612533; doi:10.1371/journal.pone.0276615)
Supplement: Attachment — Submitted filename: Reviewer comments_1_17_2022.docx [file pone.0276615.s001.docx]

Key Message:

The authors provide an extensive and thorough retrospective case series of a single specialty facility experience of ventricular septal rupture post-MI in a low-middle income country. They provide a much-needed perspective on the management of a rare but often catastrophic post-MI complication which carries an exceptionally high mortality rate, particularly in those who are unable to undergo surgery. They concluded that a SCAI shock classification of C, D, or E at presentation was a strong predictor of mortality and that determining the optimal time for surgical intervention remains a complex problem to solve. Noting that while patients who have delayed surgery have better survival rates, a strong survivorship bias exists. They further included long-term health-related quality-of-life data, attained from survivors. They should be applauded for their hard work.

Major Points:

1. Results section 2.9 Predictors of Mortality (page 10, line 200): non-surgical management is referred to as a predictor of hospital mortality. In the review of the clinical characteristics of these 31 patients reviewed elsewhere in the paper – 12 were initially intended to go to surgery but rapidly deteriorated within 12 hours of this decision and died prior to surgery. In another 16, patients were considered prohibitive or poor candidates for surgery by the medical team. In the final 3, the family declined invasive measures. With the above in mind, it was not necessarily the decision to pursue non-surgical management that led to their demise, but rather clinical and demographic characteristics that made them poor or prohibitive candidates for surgery that led to poor clinical outcomes. This should be clarified and elaborated on in the text.
2. From Table 1, 87% of patients in the non-surgically managed patients had class C, D, or E shock and only 29% of all patients in the non-surgically managed group received mechanical support in the form of an IABP. Comparatively, in the surgically managed group 69% had stage C, D, or E shock and 61% of all patients in the surgically managed group received an IABP. Similarly, 42% of patients in the surgically managed group received reperfusion compared to only 26% in the non-surgically managed group. Are you able to tease out exactly what led to decreased use of MCS/IABP and reperfusion in the non-surgically managed group? Was there some common clinical feature that made patients in this group to be less favorable candidates for MCS, reperfusion, and surgery? Co-morbid conditions? Were there gender differences? Would suspect time-to-presentation to be a driver of this, however per Table 1, the median time between MI and VSR was greater in the surgically managed group compared to the non-surgically managed group.
3. Table 1, page 7: I would report the percentage of patients in the surgical treatment arm that were SCAI shock class A&B and C,D&E rather than the % of SCAI shock A&B patients in surgical treatment arm and so on (the first column of “all patients” is formatted this way while the subgroups are formatted differently). This is the same for rows: “initial reperfusion therapy”, “LV EF”, and “MR”
4. Table 1, page 7: For the rows “IABP” , “Initial reperfusion therapy” , “basal VSR” , and “RV dysfunction” – this table is meant to compare the baseline characteristics of the surgically treated and non-surgically treated groups (the test for statistical significance is to determine if the baseline characteristics of the two groups are different), with this in mind I would report the % of patients within each group that, for example, received a IABP or were revascularized rather than the distribution of patients with an IABP between the two groups. (the cumulative % of each of these rows = 100%, this should not be the case if attempting to compare baseline characteristics between the two groups).
5. Table 2, page 12: Again, this table is meant to compare the baseline characteristics of the survivors and non-survivors (the test for statistical significance is to determine if the baseline characteristics of the two groups are different), with this in mind I would report the % of patients within each group that, for example, had a VSR within 1 week of MI rather than the distribution of patients who had a VSR within 1 week of an MI between the two groups. The % of patients with a VSR within 1 week is the 2 groups is 65% in the survivors and 88% in the non-survivors, the %s displayed are 28 and 72%, respectively – this is misleading. For another example, when comparing the two groups it is more helpful to know that only 26% of the survivors were female and that 43% of the non-survivors were female. While it is helpful to know that only 24% of females survived compared to 40% of males, it is not the aim of this table.
6. Discussion (page 15, line 303): While not statistically significant in these 67 patients, only 40% of women went on to surgery compared to 62% of men and only 24% of females survived compared to 40% of males. This suggests that gender may play a role in survival however the patient population was not large enough to see a statistically significant difference.
7. Are there data regarding the coronary artery involved or distribution of ST changes on EKG? Did this correlate with the distribution of the VSR?
8. The utility of the KC Questionnaire in determining the value of surgery is a little unclear. It does suggest that appropriate surgical candidates, who undergo surgery and survive have a good subjective quality of life, but it does not necessarily suggest that poor candidates for surgery who have similar QOL.

Minor Points

**Text:**

1. Recommend language editing.
2. Recommend statistical review.
3. Introduction (page 4, line 72): Please clarify whether this is acute heart failure or a history of chronic heart failure. Later refer to high Killip class to be a risk factor for VSR.
4. Introduction (page 4, line 73): “elevated cardiac biomarkers at the time of presentation” may be clearer that “initial positive cardiac biomarkers.”
5. Introduction (page 4, line 77): is there a reference (eg autopsy study) for pre-existing coronary disease leading to increased incidence of fatal rupture?
6. Results (page 6, line 129): please clarify what is meant by the onset of the MI (e.g. the onset of symptoms? First contact with medical personnel?). This is relevant to both timing to presentation and timing to VSR. Further, in the instances in which revascularization was deferred – was this only because of timing?
7. Results, Echo and Angiographic Findings (page 8, line 142): how else was the location of the VSR characterized – there are seemingly 8 additional patients that did not have an anterior or basal VSR. Serpiginous or poorly localized?
8. Results, Echo and Angiographic Findings (page 8, line 142, 145): 19 patients corresponds to 28% not 29.2 and 28 patients corresponds to 42% not 48.3% of patients. Would recommend reviewing percentages to ensure consistency.
9. Results, Echo and Angiographic Findings (page 8, line 149): These data are not shown in Table 1.
10. Results, Management (page 8, line 160): It is stated that 16 or 46% of patients in the surgically managed group received an IABP, Table 1 reports 22 or 61% of patients received an IABP. Were some placed after or at the time of surgery? Please clarify.
11. Results, Surgical Technique (page 9, 179): Earlier in the text you refer to 1 patient who underwent percutaneous closure, in this you refer to ‘both’ patients – was there a patient who underwent surgery who later underwent percutaneous closure as well? Please clarify.
12. Results, Mortality (page 10, 187): while Figure 2 demonstrates the exceptionally high rate of mortality in the immediate days after diagnosis of VSR, this paragraph is convoluted. Namely, the incident rate of mortality in the surgical intervention group is low because some patients survived for a long time, rather than there being a low mortality rate immediately following surgery. May be worth plotting the KM curve of the non-surgical vs surgically managed group in Figure 2 to illustrate the overwhelmingly high mortality in non-surgically managed patients.
13. Results, Predictors of Mortality (page 10, 205): was there a difference in outcomes in patients who received MCS specifically in patients with SCAI stage C, D, or E cardiogenic shock?
14. Results, Predictors of Mortality (page 10, 208): was there a difference in outcomes in patients who received PCI vs thrombolytics? Ie was the poorer outcome seen with intervention consistent across patients who received PCI as well as thrombolytics?
15. Discussion (page 13, line 255): Would be cautious to use the terminology of “early presentation” was a predictor of early mortality; it seems rather that “early VSR after MI” was a predictor, not early presentation in general.
16. Discussion (page 14, line 295): Again, would be cautious to use the terminology of “early presentation” but rather “early VSR after MI” – further would be cautious to say early surgery is a predictor of mortality. This is likely due to survivorship bias, as you explained later in the discussion.
17. Discussion (page 15, line 309): It states a large portion of the patient population (81%) presented early after MI – however revascularization was deferred in 66% of patients due to timing. Please clarify this.
18. Discussion (page 16, line 326): This is hard to follow, please clarify what you be the most clinically appropriate approach eg early vs delayed strategy.
19. Discussion (page 17, line 349): Was revascularization attempted in the 24-48 hr range in select patients in this study?

**Tables and Figures:**

1. Table 1, page 7: Recommend keeping decimal points consistent between mean and SD.
2. Table 1, page 7: If reporting significance in Table 1, would recommend reporting p-values rather than “NS” (alternatively, could delete column of p-values altogether and use footnotes to denote statistically significant differences) – being a retrospective case series of a relatively small number of patients (67), it was not necessarily powered to detect differences in these categories. For example, 62% of men went for surgery compared to only 40% of women. While this may have occurred by chance, it is worth further investigation regardless of the p-value. (e.g. it is possible the women were older? Given that in the non-surgical treatment arm, patients were on average older and there was a higher percentage of women in this group.)
