# Supplementary material for: Management and outcome of post-myocardial infarction ventricular septal rupture—A Low-Middle-Income Country Experience
Source: PLoS One. 2022 Oct 27;17(10):e0276615. doi: 10.1371/journal.pone.0276615 (PMC9612533; doi:10.1371/journal.pone.0276615)
Supplement: Attachment — Submitted filename: RESPONSE TO THE REVIEWERS COMMENTS.docx [file pone.0276615.s003.docx]

**RESPONSE TO REVIEWERS’ COMMENTS**

Thank you for a detailed review that help in improving our paper. Here are the responses for minor revisions as asked.

**Additional Editor Comments (if provided)**

**1.** The authors should replace Figure 2 with a Kaplan-Meier plot showing the surgical and non-surgical groups along with an appropriate figure legend.

*Updated Figure 2 has been uploaded with appropriate legend.*

**Reviewer #2:**

**1.** I do not think the KM curve, as presented, adds much to the paper because it only includes one arm (maybe all comers?) and the time scale is not as help as say a 30-day of 6-month time scale would be.

*The KM curve is reconstructed using 2 groups with revised scale, updated figure 2 is uploaded.*

**Reviewer #3:**

**1.** Figure 2 is a KM plot of the entire cohort and the text indicates that there were differences between patients undergoing intervention versus those that did not. The authors should replace this figure with a KM plot of the two groups. The plot that they included in the response to the reviewers, however, only has 59 subjects, not 67. The authors need to correct this.

*The KM curve is reconstructed using 2 groups with revised scale, new figure 2 is uploaded with N=67.*

**2.** The authors should include figure legends, especially for figure 2.

*Figure legends are added to the manuscript.*
